# Factors Affecting Medical Students' Uptake of the 2009 Pandemic Influenza A (H1N1) Vaccine

**DOI:** 10.1155/2012/753164

**Published:** 2012-11-28

**Authors:** Siang I. Lee, Ei M. Aung, Ik S. Chin, Jeremy W. Hing, Sanghamitra Mummadi, Ghunavadee D. Palaniandy, Rachel Jordan

**Affiliations:** ^1^College of Medical and Dental Sciences, University of Birmingham, Edgbaston, Birmingham B15 2TT, UK; ^2^Unit of Public Health, Epidemiology and Biostatistics, School of Health and Population Sciences, College of Medical and Dental Sciences, Public Health Building, University of Birmingham, Edgbaston, Birmingham B15 2TT, UK

## Abstract

*Background*. Pandemic influenza vaccination rate amongst healthcare workers in England 2009/2010 was suboptimal (40.3%). Targeting medical students before they enter the healthcare workforce is an attractive future option. This study assessed the H1N1 vaccine uptake rate amongst medical students and factors that influenced this. *Methods*. Anonymised, self-administered questionnaire at a medical school. *Results*. The uptake rate amongst 126 medical students offered the vaccine was 49.2% and intended uptake amongst 77 students was 63.6%. Amongst those offered the vaccine, the strongest barriers to acceptance were fear of side effects (67.9%), lack of vaccine information (50.9%), lack of perceived risk (45.3%), and inconvenience (35.8%). Having a chronic illness (OR 3.4 (95% CI 1.2–10.2)), 4th/5th year of study (OR 3.0 (95% CI 1.3–7.1)), and correct H1N1 knowledge (OR 2.6 (95% CI 1.1–6.0)) were positively associated with uptake. Non-white ethnicity was an independent negative predictor of uptake (OR 0.4 (95% CI 0.2–0.8)). Students who accepted the H1N1 vaccine were three times more likely (OR 3.1 (95% CI 1.2–7.7)) to accept future seasonal influenza vaccination. *Conclusion*. Efforts to increase uptake should focus on routine introduction of influenza vaccine and creating a culture of uptake during medical school years, evidence-based education on vaccination, and improving vaccine delivery.

## 1. Introduction

The novel influenza A (H1N1) outbreak was declared a pandemic by the World Health Organisation (WHO) on 11th June 2009 [1]. In the United Kingdom (UK), 474 deaths had occurred by the end of the 2009/10 influenza season with the highest case-fatality rate among the over 65-year age group [1, 2]. Vaccination is one of the intervention strategies used to mitigate an influenza pandemic [3, 4]; therefore, as part of the Department of Health (DH) vaccination policy healthcare workers were recommended to receive the H1N1 vaccine to protect themselves, protect patients, and maintain frontline services during the pandemic [5, 6]. At the University of Birmingham, clinical year (3rd–5th year) medical students were also offered the H1N1 vaccine from November 2009 [7].

Influenza vaccination of health care workers (HCW) is known to be effective in preventing seasonal influenza, reducing absenteeism, and protecting patients against nosocomial infection with a resultant decrease in morbidity and mortality [6, 8]. In spite of these benefits, seasonal influenza vaccine uptake among HCW in the UK has traditionally been disappointingly low with uptake of less than 20% in the pre-pandemic year of 2008/2009 in the UK [9]. Similarly low uptake rates have been reported in many other European countries [10]. During the pandemic year, there was a slight increase of HCW seasonal influenza vaccine uptake from 16.5% to 26.4%, while pandemic influenza vaccine uptake among HCW reached 40.3% in the UK [9].

However, vaccine uptake amongst HCW remains suboptimal. It has been frequently found that previous history of seasonal influenza vaccination is a strong predictor of both seasonal and H1N1 vaccine uptake [11–15]. Although no studies have shown a correlation between H1N1 vaccine uptake with subsequent seasonal influenza uptake, figures in England showed an increase in the latter after the H1N1 pandemic [16]. Achieving a high vaccine uptake in the early stages of a medical career might therefore improve subsequent influenza vaccine uptake as suggested by Amodio et al. [17]. Previous studies have shown suboptimal influenza uptake amongst medical students (USA 48% [18], Hong Kong 67% [19]). Since medical students in the UK are not routinely offered the influenza vaccine and nor are there data on their influenza vaccine uptake, the pandemic situation allowed us to determine H1N1 influenza vaccine uptake in this group and identify key factors influencing this. As they are HCW of the future, maximising uptake by addressing barriers in this population may help improve subsequent uptake rates.

## 2. Materials and Methods

### 2.1. Study Design

This was a cross-sectional study carried out between February and March 2010 using an anonymised self-completed questionnaire.

### 2.2. Subjects and Setting

The sample population consisted of 481 out of more than a thousand medical students from the University of Birmingham. 40 subjects were Graduate Entry Course 1 (GEC1) students, 70 were from year 2, 194 from year 3, 60 from year 4 and 117 from year 5. This population was selected and allocated randomly by study group by the Project Scrutiny Committee of the College of Medical and Dental Sciences, University of Birmingham. Initially, a link to the questionnaire was emailed to the allocated sample with a reminder sent after a week. The web-based survey was open for two weeks. Owing to a poor response rate using this method, paper questionnaires were subsequently distributed to the originally allocated sample and to a convenience sample of students that were on similar hospital placement as the authors. These were distributed to the selected year 2 students during their tutorials and to years 3–5 students at their respective hospital placements.

### 2.3. Influenza Immunisation Programme

Medical students were made aware of the availability of the pandemic influenza vaccine via notices posted on the student notice boards, emails from the hospitals where students were placed and a general email from the medical school. The vaccine was not formally offered to the medical student cohort as a whole, rather it was offered opportunistically by some hospital trusts depending on each hospital's policy.

The hospital trusts allocated a set date, time, and location where medical students could receive the vaccination from trained staff during their hospital placements. Seasonal influenza vaccination was offered simultaneously. Both vaccines were available free of charge.

### 2.4. Questionnaire

The questionnaire (see the appendix) collected sociodemographic data (age; sex; year of study; history of chronic illness; living with specified groups including children under 16, pregnant woman, over 65's, and healthcare workers), information regarding smoking status, previous seasonal influenza vaccination and adverse reactions, perceived severity of swine flu, reasons for and against H1N1 immunisation, influences affecting immunisation, and three knowledge items related to swine flu. Reasons for and against vaccination were adapted from questionnaires used in previous papers [12, 20, 21]. Participants were prompted to rank three reasons for acceptance or declination and three major influences. Since a significant number of respondents failed to rank their responses, we did not analyse the result according to rank. The questionnaire was piloted prior to the study.

### 2.5. Outcomes

The primary outcome was receipt of H1N1 vaccine, but for those not offered H1N1 vaccine, there was an option to reply with “intention to vaccinate”.

### 2.6. Statistical Analysis

Analysis was performed using Statistical Package for the Social Sciences (SPSS) v19. Basic descriptive statistics were performed. In order to identify factors significantly associated with H1N1 uptake, odds ratios and 95% CIs were calculated using binary logistic regression among those offered the vaccine. Factors with a *P*  value < 0.05 and clinical factors known to be associated with vaccine uptake (sex, ethnicity, chronic illness, and year of study) were then entered into multiple logistic regression analysis. Due to small numbers, smoking status was re-categorised into “never” and “ever” smokers and ethnicity as “White” and “Non-white”. For the perception of H1N1 severity question, “Quite severe”, “Severe” and “Very severe” were merged together as “severe.”

## 3. Results

### 3.1. Response Rate

We received 31 responses as a result of the initial email. After the reminder, the total number of respondents was 68 (14.1% response rate). After the paper-based approach, where 481 questionnaires were distributed, 137 (28.5%) were returned. The data from both methods were combined for analysis (205, 42.6%). Respondents with incomplete H1N1 vaccination status (2/205) were excluded. In total, 203 (42.2%) entries were included in the study.

### 3.2. Characteristics of the Survey Respondents

The characteristics of the survey respondents are shown in Table 1. Of the 203 participants, 126 respondents were offered the vaccine whilst 77 were not. The majority were females [*n* = 148 (72.9%)], and in their third year of medical school [93, [45.8%]]. Previous uptake of seasonal influenza vaccine was low and amongst those who had received vaccine, 20 (28.2%) had suffered side effects. Patterns were largely similar between those who were and were not offered the vaccine, but those offered were largely from year 3 and over, were more likely to be Asian, have a chronic illness and have had seasonal influenza vaccination that season or previous seasons.

### 3.3. H1N1 Influenza Vaccination: Intended and Actual Uptake

Of the 126 respondents who were offered the H1N1 vaccine, 62 (49.2% (95% CI 40.2–58.3%)) accepted the vaccine. Of the 77 not offered, 49 (63.6% (95% CI 51.9–74.3%)) stated they would accept the vaccine if offered. Those not offered were significantly more likely (*P* = 0.046) to state that they would accept the vaccine compared to those offered (OR 1.8 (95% CI 1.01–3.2)).

### 3.4. Reasons for Acceptance or Declination of Influenza Vaccine

**Figure 1:**
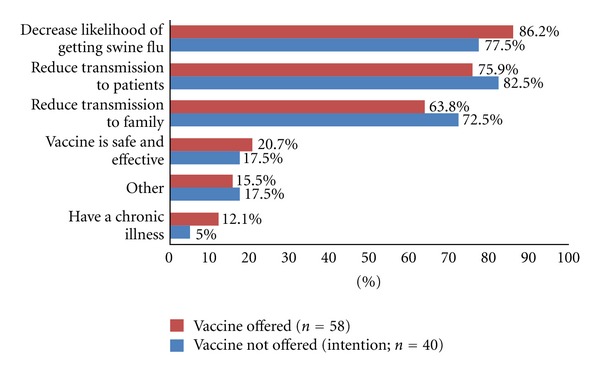
Main reasons for H1N1 vaccine acceptance/intention to be vaccinated among medical students.

The three most frequently quoted reasons given for acceptance amongst both those offered and not offered the vaccine (although in a slightly different order) were to decrease the likelihood of getting H1N1 (86.2% (50/58) and 77.5% (31/40), respectively), reduce transmission to patients (75.9% (44/58) and 82.5% (33/40)), and to decrease the likelihood of transmission to family members (63.8% (37/58) and 72.5% (29/40)), (Figure 1). 

**Figure 2:**
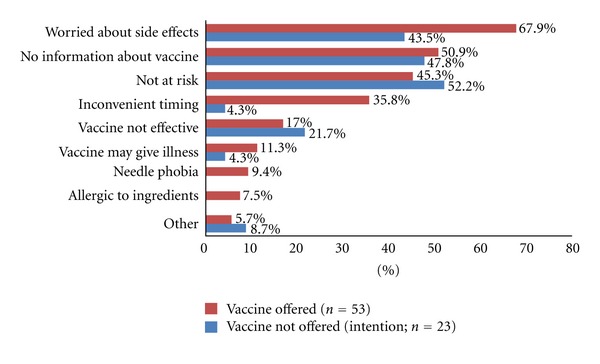
Main reasons for H1N1 vaccine declination/intention to decline among medical students.

The main reason given for declination of the vaccine amongst those offered was worry about side effects (67.9% (36/53)), followed by lack of information about vaccination (50.9% (27/53)) and perceiving they were not at risk (45.3% (24/53)), (Figure 2). However, a further key reason for having been offered but not actually having received the vaccination was “inconvenient timing” (35.8% (19/53)). For those who were not offered the vaccine, the main reasons for intention to decline were perception that they were not at risk (52.2% (12/23)), lack of information (47.8% (11/23)) and worry about side effects (43.5% (10/23)), (Figure 2).

**Figure 3:**
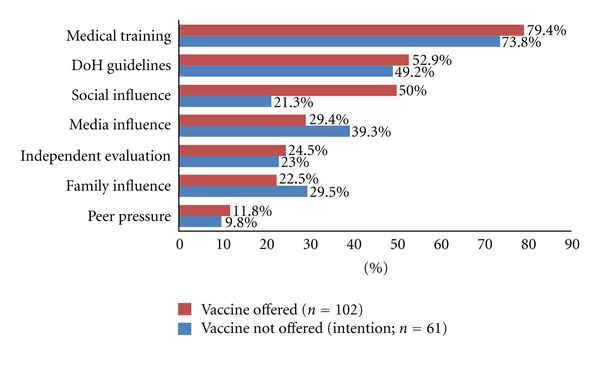
Extrinsic factors affecting H1N1 vaccine uptake/intention to be vaccinated among medical students.

The three major reported extrinsic influences affecting actual H1N1 vaccine uptake were medical training (79.4% (81/102)), DH recommendations (52.9% (54/102)) and social influences (50.0% (51/102)). For those who had not been offered the vaccine, they also reported medical training and DH guidelines as important (73.8% (45/61) and 49.2% (30/61), resp.), although the third most important influence cited was the media (39.3% (24/61)) (Figure 3).

### 3.5. Knowledge Regarding H1N1 Pandemic

Table 2 describes the students' knowledge and attitudes about the swine flu epidemic. Only a third of the respondents correctly identified all the priority groups (35.5%), and only 23.2% correctly identified methods of H1N1 virus transmission. Almost half of participants (59 (46.8%)) thought the swine flu epidemic was not severe. Level of knowledge and perceived severity were similar across both the students offered and those not offered the vaccine. 

### 3.6. Determinants of H1N1 Vaccination Uptake

Table 3 presents the factors which affected actual vaccine uptake amongst those offered the vaccine. On univariate analysis, there was no significant difference in uptake between the sexes, amongst smokers compared with ever smokers, or among students living with “at risk” groups. Students of non-white ethnicity were significantly less likely to take up the vaccine (OR 0.4 (95% CI 0.2–0.8)), although those reporting a chronic illness were more likely to do so (OR 3.4 (95% CI 1.2–10.2)). Students in years 4 and 5 were more likely to receive the vaccine (OR 3.0 (95% CI 1.3–7.1)), and those who were correct in at least one of the knowledge questions were also more likely to accept the vaccine (OR 2.6 (95% CI 1.1–6.0)).

After adjustment for sex, ethnicity, year of study, presence of chronic illness and correct knowledge answers (model 1), students of non-white ethnicity remained as an important independent negative predictor of vaccine uptake (OR 0.4 (95% CI 0.2–0.8)). Having a chronic illness (OR 3.4 (95% CI 1.2–10.2)), being in years 4 or 5 (OR 3.0 (95% CI 1.3–7.1)) and having accurate knowledge on the H1N1 pandemic (OR 2.6 (95% CI 1.1–6.0)) were positive predictors of vaccine uptake although they were of borderline significance.

The addition of previous seasonal vaccination to the multivariate model suggested that prior influenza vaccination might be a predictor of vaccine uptake, but was not statistically significant and may be explained partly by the presence of chronic illness. 

### 3.7. Future Seasonal Influenza Vaccination

When asked, students who had actually received the H1N1 vaccine were three times more likely (OR 3.1 (95% CI 1.2–7.7)) to say that they would accept the seasonal influenza vaccination in the future compared to respondents who declined the HIN1 vaccine.

## 4. Discussion

### 4.1. Main Findings of This Study

The H1N1 vaccine uptake among medical students was 49.2% and intended uptake was 63.3%. This uptake is higher than the 40.3% H1N1 vaccine uptake among frontline healthcare workers overall in England in 2009/10 although the figure is more similar to general practitioners' uptake (50.1%) [9]. It is also noted that this uptake rate is substantially higher than the seasonal influenza vaccine uptake rate amongst HCW of 16.5% in 2008/9, 26.4% in 2009/10 [9], and even the improved postpandemic levels of 34.7% in the winter season of 2010/11 [16]. Studies in other countries found H1N1 vaccine uptake rates among medical students varied between 8% in Greece [22] to 93% in Sweden [23]. 

Main barriers to uptake included fear of side effects, lack of vaccine information, lack of perceived risk, and inconvenience. Main reasons for acceptance were to protect themselves, patients, and family. The main influences on uptake were medical training, DH recommendation, and social influences (much of which would probably be from other medical personnel). Having a chronic illness, 4th/5th year of study, and correct H1N1 knowledge were associated with uptake whilst non-white ethnicity was a negative predictor. Students in years 4/5 were more likely to accept the vaccine as more years of clinical experience may have led to better understanding of the importance of vaccination and a greater sense of responsibility. This is consistent with the finding where those with better H1N1 knowledge were also more likely to accept the vaccine. Finally, H1N1 vaccine uptake predicted future seasonal influenza vaccine uptake. 

### 4.2. What Is Already Known on This Topic

Consistent with the findings of our study, many studies of seasonal and pandemic influenza vaccine have demonstrated that the most common reasons for acceptance were for self-protection and patient protection whereas the main reasons for declining were fear of side effects and doubts of efficacy [11, 24–35]. Fear of side effects, the most frequently quoted barrier to uptake of H1N1 vaccine, could be due to media interest in the association between swine flu vaccine and Guillain Barré syndrome in 1976 [36, 37], and indeed media influence played an important role in the decision making of our study group, although not the main role. Inconvenient vaccine delivery was the fourth common reason for vaccine rejection in our study which accords with a past study of healthcare workers [38]. To increase the convenience of vaccine delivery, measures such as flexible appointment times, mobile carts, and electronic reminders could be implemented [39].

In contrast to findings of other studies that showed previous seasonal influenza vaccination as a positive predictor of the pandemic influenza vaccine uptake [11–15, 40], our study did not find a significant association; this is most likely because few students would have been offered the vaccine in the past except for those few with a chronic illness such as asthma. 

Some studies have shown that ethnic minorities, particularly the black ethnic group, are less likely to accept the H1N1 or seasonal influenza vaccine as they are most likely to perceive the vaccine as unsafe [41–44]. Galarce et al. found that in the USA, blacks were most likely to have tried getting the H1N1 vaccine but found it unavailable [44]. There is little other data on uptake amongst South Asians, which is the most prevalent ethnic minority in our study and non-white ethnicity was shown to be a negative predictor of H1N1 vaccine uptake in our study.

### 4.3. What This Study Adds

As far as we are aware, this study is the first to report H1N1 vaccination uptake among medical students in the UK. As such, it sets the scene for potential future influenza vaccination campaigns to begin during medical school. 

Uptake/intention to receive H1N1 vaccine amongst medical students appears to be higher than that of HCW (40.3%) [9], although this is still below optimal levels. The barriers mentioned could be overcome by education about the efficacy and safety of the vaccine and reassurance that side effects are infrequent and usually mild. The formal medical training environment and guidance from the DH are clearly cited as the most important influencing factors and the medical course is a prime opportunity to exploit this, perhaps particularly in years 4 and 5. It is also important to increase awareness that medical students are at greater risk of contracting H1N1 due to the nature and extent of their patient contact during placements. Informing students about the potential for transmission to patients should also become a priority in the effort to improve uptake. This is an important aim of interventions as our findings show that even a minimal knowledge about H1N1, its severity and transmission can increase uptake significantly. 

Our study showed that subjects who accepted the H1N1 vaccine were three times more likely to state they would accept future seasonal influenza vaccines. This reinforces the importance of taking actions to increase H1N1 vaccine uptake amongst medical students. Acceptance of both pandemic and seasonal influenza vaccine is proven to be strongly associated with previous seasonal influenza vaccine uptake among HCW later in their careers [13, 14]. Hence, efforts to create a culture of seasonal influenza vaccination during student years are likely to impact on vaccine uptake in later years. Mandatory vaccination of healthcare workers against influenza is increasingly being called for on both moral and professional ethical grounds [45]. Perhaps medical school is the place to start. 

### 4.4. Limitations of This Study

Valid responses were received from 201 students, who although were randomly selected, may not reflect the views and responses of all medical students in the UK. There were more year 3 respondents, which maybe because students from the same year as the authors (year 3) may be more willing to respond to the questionnaire. Many respondents failed to rank their responses to the reasons for uptake/declination which resulted in our data analysis changing from a weighted ranking as originally planned to a tally of reasons. However this data analysis approach is consistent with other published studies.

We attempted to establish any association between knowing someone who had contracted H1N1 and vaccine uptake. A significant majority (143 (70.4%)) answered yes. This question could have focused specifically on first person contact and explored further how this could influence vaccination.

## Figures and Tables

**Table 1:** Characteristics of respondents.

Factor		Vaccine offered group, *n* = 126 (%)	Vaccine not offered group, *n* = 77 (%)	Total *n* = 203 (%)
Sex	Male	34 (27.0)	20 (26.0)	54 (26 .6)
	Female	92 (73.0)	56 (72.7)	148 (72.9)
	Missing data		1 (1.3)	1 (0.5)

Age (mean (SD))		21.5 (1.8)	21.2 (2.4)	21.4 (2.1)
	Missing data	2	2	4

Ethnicity	White	77 (61.1)	63 (81.8)	140 (69.0)
	Asian	28 (22.2)	9 (11.7)	37 (18.2)
	Chinese	11 (8.7)	2 (2.6)	13 (6.4)
	Mixed	2 (1.6)	0 (0)	2 (1.0)
	Other	7 (5.6)	2 (2.6)	9 (4.4)
	Missing data	1 (0.8)	1 (1.3)	2 (1.0)

Smoking status	Never smoker	116 (92.1)	69 (89.6)	185 (91.1)
	Ex-smoker	3 (2.4)	2 (2.6)	13 (6.4)
	Smoker	7 (5.6)	6 (7.8)	5 (2.5)

Chronic illness	No	107 (84.9)	70 (90.9)	177 (87.2)
	Yes	19 (15.1)	7 (9.1)	26 (12.8)
	Asthma	13 (10.3)	6^ a^ (7.8)	19^a^ (9.4)
	Other	6 (4.8)	4 (5.2)	9 (4.4)

Year of study	GEC^b^ 1	0 (0)	6 (7.8)	6 (3.0)
	Year 2	10 (7.9)	43 (55.8)	53 (26.1)
	Year 3	83 (65.9)	10 (13.0)	93 (45.8)
	Year 4	16 (12.7)	5 (6.5)	21 (10.3)
	Year 5	17 (13.5)	13 (16.9)	30 (14.8)

Lives with susceptible individuals	Under 16	7 (5.6)	2 (2.6)	9 (4.4)
	Pregnant	1 (0.8)	1 (1.3)	2 (1.0)
	Over 65	5 (4.0)	1 (1.3)	6 (3.0)
	HCW	20 (15.9)	12 (15.6)	32 (15.8)
	None of the above	95 (75.4)	62 (80.5)	157 (77.3)
	Missing data	1 (0.8)		

Seasonal influenza vaccination history	Never	67 (53.2)	61 (79.2)	128 (63.1)
	This year	35 (27.8)	5 (6.5)	40 (19.7)
	Previous years	23 (18.3)	8 (10.4)	31 (15.3)
	Missing data	1 (0.8)	3 (3.9)	4 (2.0)

H1N1 vaccine	Accept/would accept	62 (49.2)	49 (63.6)	111 (54.7)
	Decline	64 (50.8)	28 (36.4)	92 (45.3)

Knows anyone who contracted H1N1	No	32 (27.8)	28 (36.4)	60 (29.6)
	Yes	94 (74.6)	49 (63.6)	143 (70.4)

^a^3 Respondents had both asthma and other chronic illness.

^b^GEC stands for Graduate Entry Course.

**Table 2:** Knowledge and attitude of medical students towards the H1N1 pandemic.

	Knowledge questions	Respondents with correct answers, *n* (%)		
		Vaccine-offered group *n* = 126 (%)	Vaccine-not-offered group *n* = 77 (%)	Total *n* = 203 (%)
Priority groups receiving H1N1 vaccine	*Answered correctly*	45 (35.7)	27 (35.1)	72 (35.5)
	Over 65 *√* ^a^			
	Under 16			
	Pregnant women *√*			
	Healthcare workers *√*			
	People with chronic health conditions *√*			

Methods of transmission of H1N1 virus	*Answered correctly*	30 (23.8)	17 (22.1)	47 (23.2)
	Cough/sneezes *√*			
	Eating infected meat			
	Direct contact with an infected person *√*			
	Touching contaminated object *√*			

Fatality rate of H1N1	*Answered correctly*	45 (35.7)	29 (37.7)	74 (36.5)
	0.01% *√*			
	0.1%			
	1%			
	10%			
	20%			
	50%			

Attitude		*n* = 126 (%)	*n* = 77 (%)	*n* = 203 (%)

Perception of H1N1 severity	Not severe	59 (46.8)	35 (45.5)	94 (46.3)
	Quite severe/Severe/Very severe	67 (53.2)	42 (54.6)	109 (53.7)

^a^ *√* Indicates the correct answer for the knowledge questions.

**Table 3:** Determinants of vaccine uptake in the vaccine offered group.

Variables		Numbers receiving vaccine (%)	Univariate analysis	Multivariate analysis: model 1^a^	Multivariate analysis: model 2 including term for previous seasonal vaccination^b^
Sex	Male	14 (22.6)	1.0	1.0	1.0
	Female	48 (77.4)	0.6 (0.3–1.4)	1.1 (0.4–2.6)	1.4 (0.4–4.7)

Ethnicity	White	45 (72.6)	1.0	1.0	1.0
	Nonwhite	17 (27.4)	0.4 (0.2–0.8)	0.4 (0.2–0.8)	0.3 (0.1–0.8)

Smoking status	Never smoker	58 (93.5)	1.0		
	Ever smoker	4 (6.5)	0.7 (0.2–2.5)		

Chronic illness	No	48 (77.4)	1.0	1.0	1.0
	Yes	14 (22.6)	3.4 (1.2–10.2)	3.5 (1.0–12.1)	1.8 (0.3–9.9)

Year of study	GEC 1 and	3 (4.8)	0.6 (0.1–2.3)	0.3 (0.1–1.6)	0.3 (0.02–3.2)
	Year 2	36 (58.1)	1.0	1.0	1.0
	Year 3	23 (37.1)	3.0 (1.3–7.1)	2.6 ( 1.0–6.7)	5.2 (1.6–17.4)
	Years 4 and 5				

Lives with 1 of more risk groups	No	49 (79.0)	1.0		
	Yes	13 (21.0)	0.7 (0.3–1.6)		

Seasonal influenza vaccination history	Never	22 (35.5)	1.0		1.0
	Previous years	10 (16.1)	1.6 (0.6–4.1)		3.0 (0.8–10.8)

Knows anyone who contracted H1N1	No	18 (29.0)	1.0		
	Yes	44 (71.0)	0.7 (0.3–1.5)		

Number of correct answers	None	11 (17.7)	1.0	1.0	1.0
	At least 1 correct answer	51 (82.3)	2.6 (1.1–6.0)	2.4 (1.0–6.0)	3.1 ( 0.9–11.2)

^a^Adjusted for sex, ethnicity, year, of study and knowledge.

^b^Adjusted for sex, ethnicity, year of study, knowledge, and previous seasonal influenza vaccine.

## Appendix

### Questionnaire

1. Sex: □ M  □ F
2. Age at last birthday: _________
3. Year of study: □ 1st  □ GEC1  □ 2nd
4. □ 3rd/GEC2  □ 4th/GEC3  5th/GEC4
5. Smoker: □ Current regular  □ Current occasional □ Ex-smoker
6. □ Never smoker
7. Do you have a long term medical condition? □ No
8. □ if Yes—*Please tick as appropriate below*:
9. □ Asthma  □ Diabetes  □ Epilepsy
10. □ Hypertension
11. □ Inflammatory Bowel Disease  □ Other *Please state*: ____________________
12. Do you live with anyone who is: (Please tick any that apply)
13. □ Under 16  □ Pregnant   □ Over 65
14. □ Healthcare professional
15. Have you ever had seasonal flu vaccine? (Please tick any that apply)
16. □ This year   □ Last year   □ Previous years   □ Never
17. If yes, have you had any side effects? □ No □ Yes *Please state*: ___________________
18. Would you have a seasonal flu vaccine in the future? □ Yes  □ No
19. Who do you think are the priority groups for receiving the swine flu vaccine? *Please select all relevant answers*:
20. □ Over 65  □ Under 16  □ Pregnant women   □ Healthcare workers □ People with chronic health conditions
21. How do you think swine flu is transmitted?
22. *Please select all relevant answers*:
23. □ Coughs/Sneezes   □ Eating infected meat
24. □ Direct contact with an infected person
25. □ Touching a contaminated object   □ Other *Please state*: ____________________
26. How severe do you think is the swine flu pandemic?
27. □ Not severe   □ Quite severe  □ Severe
28. □ Very Severe
29. What do you think is the swine flu case-fatality rate? □ 0.01%  □ 0.1%  □ 1%  □ 10%
30. □ 20%  □ 50%
31. Do you know anyone who had swine flu?
32. □ Yes   □ No
33. Have you been offered the swine flu vaccine?
34. □ Yes, and I accepted the vaccine   □ Yes, but I declined the vaccine
35. □ No, but I will have the vaccine   □ No, and I will not have the vaccine
36. If you have accepted or will accept the vaccine, why? *Please rank the 3 most relevant reasons in order, 1 being most important *
37. □ Reduce transmission to patients  □ Reduce transmission to relatives/friends  □ Decrease the likelihood of getting swine flu   □ Vaccine is safe and effective   □ I have a chronic condition   □ Other *Please state*: ____________________
38. Which source of information influenced your decision on vaccination the most? *Please rank the 3 most relevant reasons in order, 1 being most important *
39. □ Social influence  □ Media influence
40. □ Department of Health guidelines  □ Peer pressure   □ Family  □ Medical training
41. □ Independent evaluation of scientific papers
42. If you have declined or will decline the vaccine, why?* Please rank the 3 most relevant reasons in order, 1 being most important *
43. □ Do not think myself at risk of swine flu  □ Lack of information about the vaccine  □ Do not think the vaccine is effective  □ Needle phobia  □ Worried about the side effects  □ Timing of vaccination was inconvenient  □ Vaccine might give me swine flu  □ Allergies to constituents of vaccine  □ Other *Please state*: ______________________
44. Ethnicity: Please circle as appropriate if selecting “other” please state:

**Table:**

White	Asian	Black	Mixed	Other ethnicities
White British	Indian	Caribbean	White/Black Caribbean	Chinese
White Irish	Pakistani	African	White/Asian	Other groups
Other	Bangladesh	Other Black	Other mixed	
	Other Asian			
